# Bioinformatics analysis and validation of mesenchymal stem cells related gene MT1G in osteosarcoma

**DOI:** 10.18632/aging.205809

**Published:** 2024-05-13

**Authors:** Sikuan Zheng, Xifu Cheng, Sulun Ke, Linyi Zhang, Hui Wu, Dingwen He, Xigao Cheng

**Affiliations:** 1 The Second Affiliated Hospital of Nanchang University, Nanchang, China; 2 The Second Clinical College, Medical College of Nanchang University, Nanchang, China; 3 Institute of Orthopedics of Jiangxi Province, Nanchang, China; 4 School of Ophthalmology and Optometry, Nanchang University, Nanchang, China; 5 Nanchang University Queen Mary School, Jiangxi Medical College of Nanchang University, Nanchang University, Nanchang, China

**Keywords:** osteosarcoma, MT1G, mesenchymal stem cells, prognosis

## Abstract

Background: Osteosarcoma (OS) is a primary malignant bone tumor arising from mesenchymal cells. The standard clinical treatment for OS involves extensive tumor resection combined with neoadjuvant chemotherapy or radiotherapy. OS's invasiveness, lung metastasis, and drug resistance contribute to a low cure rate and poor prognosis with this treatment. Metallothionein 1G (MT1G), observed in various cancers, may serve as a potential therapeutic target for OS.

Methods: OS samples in GSE33382 and TARGET datasets were selected as the test cohorts. As the external validation cohort, 13 OS tissues and 13 adjacent cancerous tissues from The Second Affiliated Hospital of Nanchang University were collected. Patients with OS were divided into high and low MT1G mRNA-expression groups; differentially expressed genes (DEGs) were identified as MT1G-related genes. The biological function of MT1G was annotated using Kyoto Encyclopedia of Genes and Genomes (KEGG), Gene Ontology (GO) and gene set enrichment analysis (GSEA). Gene expression correlation analysis and competing endogenous RNA (ceRNA) regulatory network construction were used to determine potential biological regulatory relationships of DEGs. Survival analysis assessed the prognostic value of MT1G.

Results: MT1G expression increased in OS samples and presented higher in metastatic OS compared with non-metastatic OS. Functional analyses indicated that MT1G was mainly associated with spliceosome. A ceRNA network with DEGs was constructed. MT1G is an effective biomarker predicting survival and correlated with increased recurrence rates and poorer survival.

Conclusions: This research identified MT1G as a potential biomarker for OS prognosis, highlighting its potential as a therapy target.

## INTRODUCTION

Osteosarcoma (OS) is the predominant malignant bone tumor in clinical practice. With the widespread implementation of multidrug chemotherapy strategies in the 1970s, the five-year survival rate of patients with conventional high-grade OS has significantly improved (60-70%) [1]. Unfortunately, due to the stagnation of research progress in related fields, the survival rate has not improved since then [2], and the risk of adverse toxic events poses a great obstacle in the development of new treatment strategies. Therefore, further studies on the molecular mechanisms of OS progression are still needed to reveal opportunities for new therapeutic strategies to improve the survival rate of OS patients. Existing studies have demonstrated that OS exhibits a complex karyotype with a high degree of genetic and chromosomal instability reflected by multiple rearrangements across the genome, kataegis and chromothripsis [3–6]. Extensive reports have described genetic markers associated with prognosis [7, 8], and existing evidence indicates that transcriptional programs in OS may be epigenetically regulated [9–13]. This provides a theoretical basis for the formulation of new strategies for cancer treatment [14].

Cancer recurrence and metastasis are key factors contributing to the poor treatment outcomes in OS [15]. However, existing research has failed to elucidate the specific molecular mechanisms underlying the proliferation and invasion of OS cells. Previous studies have indicated that stromal cells, including mesenchymal stem cells (MSCs), in the tumor microenvironment can influence the proliferation and invasion of cancer cells by generating a series of signaling factors [16, 17]. Studies have successfully demonstrated that the transformation of MSCs into OS cells can occur through aneuploidy and genome loss, suggesting a potential derivation of OS cells from BMSCs [18]. Moreover, several studies have indicated that the occurrence of ferroptosis in MSCs significantly impacts tumor progression, with MT1G emerging as a critical regulator of this process [19, 20]. Nevertheless, the role of MT1G in OS remains unclear. The purpose of this study was to further elucidate the pathogenesis of OS and investigate the role of bone BMSCs in OS proliferation and invasion. We obtained transcriptome sequencing data from 91,439 cells isolated from primary and metastatic samples obtained from patients. Our study indicated low expression of metallothionein 1G (MT1G) as a potential biomarker associated with a poor prognosis in OS. The gene set enrichment analysis (GSEA) results indicated that MT1G was mainly associated with spliceosome. We also demonstrated the feasibility of MT1G as a biomarker in OS samples, providing a potential strategy for clinical OS treatment.

## MATERIALS AND METHODS

### Acquisition of cell samples and processing of single-cell RNA-seq data

We downloaded the single-cell transcriptome expression profiles of 129755 cells raw data in 11 samples from GSE152048 via the Gene Expression Omnibus (GEO) database (https://www.ncbi.nlm.nih.gov/geo/). The MSCs from primary and metastasis samples were finally analyzed in our study after filtering out poor-quality cells.

We generated a Seurat object based on the transcriptome sequencing data using the Seurat package [21]. We excluded cells with mitochondrial genes accounting for over 10% of the total gene expression and extracted transcriptome sequencing data from 91439 cell patient-derived primary and metastatic samples. The top 2000 genes with highly variable features accounting for cell-to-cell differences were identified by variance analysis and subjected to data scaling and centering. These variable genes were further used for principal component analysis (PCA) with linear dimensionality reduction. The top 35 principal components (PCs) were applied for graph-based clustering (res = 0.4) to identify distinct groups of cells. The cell clusters were visualized based on the UMAP method of dimensionality reduction. Clusters were annotated through the well-known cellular marker genes. Differentially expressed genes (DEGs) of MSCs between metastasis tissue and primary tissue were screened with pval.adj =0.05 as the cutoff criterion.

### Bulk transcriptomic data sets

We acquired gene expression records from GSE33382 (GEO), comprising 84 OS patients and 3 normal controls. Using the median value of MT1G expression as the dividing line, the 84 patients were separated into groups with high (n = 42) and low (n = 42) MT1G mRNA expression. The limma (http://www.bioconductor.org/packages/release/bioc/html limma.h1ml) package in R version 3.6.3 (http://R-project.org) was used to identify prominent DEGs between the high and low MT1G-expression groups. When the p-value was less than 0.01, genes were considered to have differential expression. The R package pheatmap (https://cran.r-project.org/web/packages/pheatmap/index.html), version 1.0.12, was used to create heatmaps. In addition, gene expression profiles and clinical data were extracted from TARGET (https://ocg.cancer.gov/programs/target). Using the median value as the dividing line, the TARGET cohort (n = 88) was split into high/low groups (high, 44 cases; low, cases). The same approach as previously reported was utilized to identify DEGs between the high and low TARGET groups. The DEGs shared by the GSE33382 and TARGET data sets were shown using a Venn diagram made in ggplot2 (https://cran.r-project.org/web/packages/ggplot2/index.html).

### Tissue samples and immunohistochemistry (IHC)

The Second Affiliated Hospital of Nanchang University provided 13 OS tissues and 13 adjacent cancerous tissues from January 2019 to December 2021 as an external validation cohort. Samples were provided as formalin fixed paraffin-embedded blocks. This research was approved by the Ethics Committee of The Second Affiliated Hospital of Nanchang University [Review (2018) No. (107)]. All participants provided informed consent. A total of 13 OS tissues and 13 adjacent cancerous tissues were included in this study. A tissue microarray was created from these 26 tissues and was used for IHC staining. Histochemical scores were calculated using the Quant Center analysis tool. The formula was calculated as follows: H score= ∑(PI × I) = (% weak intensity cells × 1) + (% moderate intensity cells × 2) + (% high intensity cells × 3) [22].

### Functional enrichment analysis

The enrichment analysis of DEGs in the MT1G high/low expression groups in the test cohort was performed by Kyoto Encyclopedia of Genes and Genomes (KEGG) and GO (Gene Ontology). Significant changes in function and pathways between the high/low MT1G expression groups were identified using GSEA and the reference gene set used was c2.cp.v7.2.symbols.gmt (Curated). Data visualization was performed using the ggplot2 package.

### Construction and hub gene extraction

We completed the protein-protein interaction (PPI) network construction of DEGs using the STRING online tool (https://cn.string-db.org) and further used Molecular Complex Detection (MCODE, http://apps.cytoscape.org/apps/mcode), Cytoscape version 3.6.2) to screen the core proteins in the PPI network.

### Correlation analysis and competing endogenous RNA (ceRNA) network construction

Spearman’s correlations were calculated to determine the association between MT1G expression in the TARGET cohort and the expression of 12 DEGs (CCND1, CDKN2A, HES1, MRPS18C, MT1E, MT1F, MT1M, MT1X, MT2A, PRPF3, PRSS27, TRIB2) common between the GSE33382 and TARGET data sets. R software and ggplot2 were used to visualize the data.

Then, the limma package was used to obtain the differentially expressed long noncoding RNAs (DELs) from the TARGET cohort. Finally, the miRcode database (http://www.mircode.org) was used to predict highly conserved microRNAs (miRNAs) associated with DELs. Meanwhile, we used the miRDB (http://mirdb.org/), miRTarBase (http://miRTarBase.cuhk.edu.cn/) and TargetScan databases (http://www.targetscan.org/mamm_31/) to predict downstream target genes of miRNAs. The common DEGs in the comparison results were used to establish the ceRNA network.

### Survival analysis and diagnostic performance assessment

Kaplan-Meier (KM) analysis of the TARGET data was performed by using the R packages survival (https://www.rdocumentation.org/packages/survival/versions/2.42-3) and survminer (https://cran.rstudio.com/web/packages/survminer/index.html). Subsequently, the timeROC package (https://cran.r-project.org/web/packages/timeROC/index.html) was used to analyze the survival prognosis of MT1G high expression patients at 1, 3 and 5 years.

We used the pROC package (https://cran.r-project.org/web/packages/pROC/) to generate receiver operating characteristic (ROC) curve with vertical coordinates for true positive rate/sensitivity and horizontal coordinates for false positive rate/specificity. To determine whether MT1G could be used as a biomarker to determine the difference between OS and normal tissues, the cutoff value that gave the highest likelihood ratio was selected.

### Statistical analysis

GraphPad Prism 8 software was used to compare expression data with one-way ANOVA and two-tailed Student’s t tests. Each experiment was conducted at least three times, and all results were expressed as the mean standard deviation (SD). The relevance of the statistics was described as follows: n.s., not significant.

### Availability of data and materials

We used the databases GSE33382 and GSE152048. The datasets for this study can be found in the Gene Expression Omnibus (GEO) database (https://www.ncbi.nlm.nih.gov/geo/). This is an open-source database.

## RESULTS

### Processing of single-cell RNA-seq profiles and screening of MSC-associated marker genes

**Figure 1:**
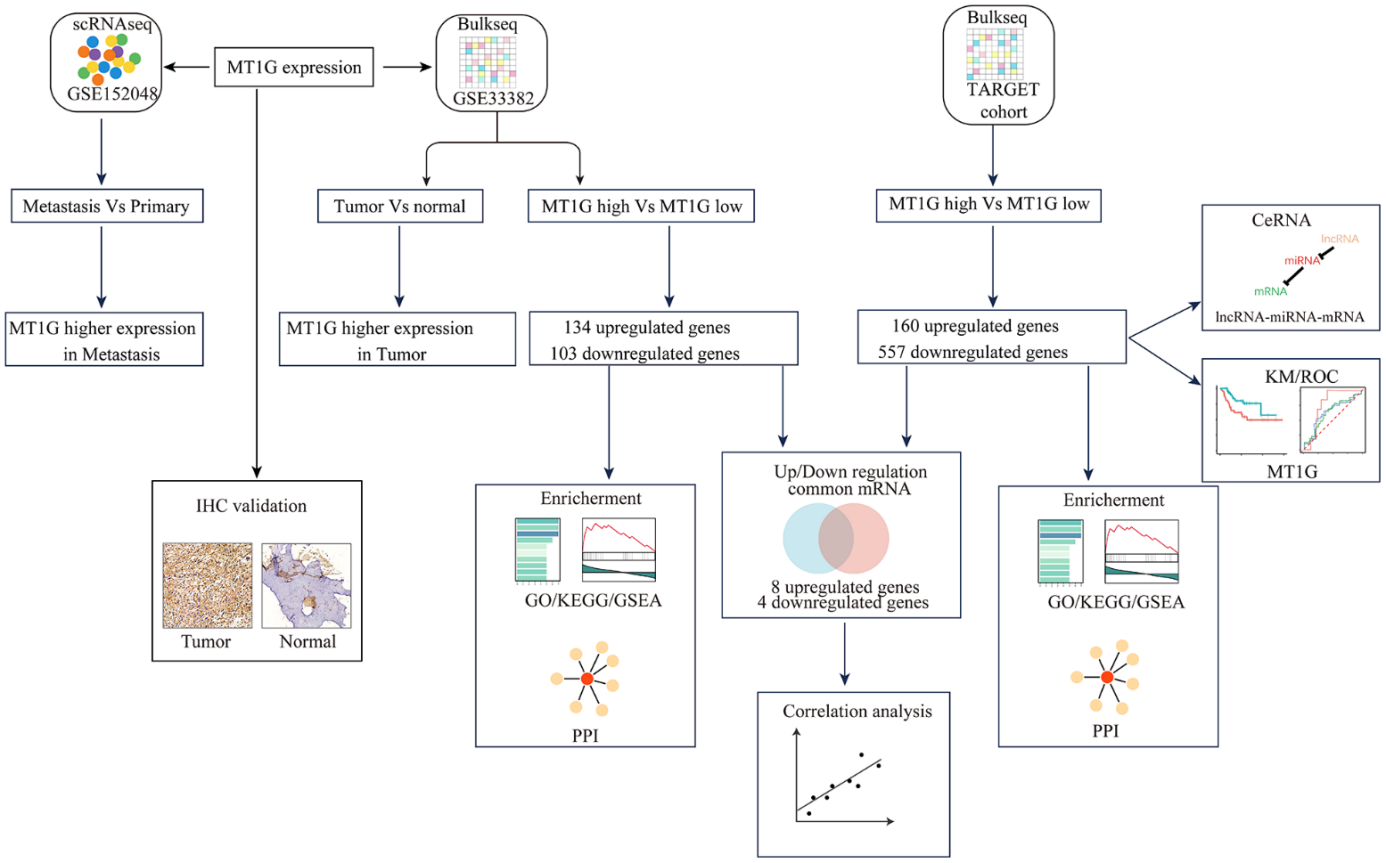
**Research flowchart.** The research process of this study is depicted in Figure 1.

**Figure 2:**
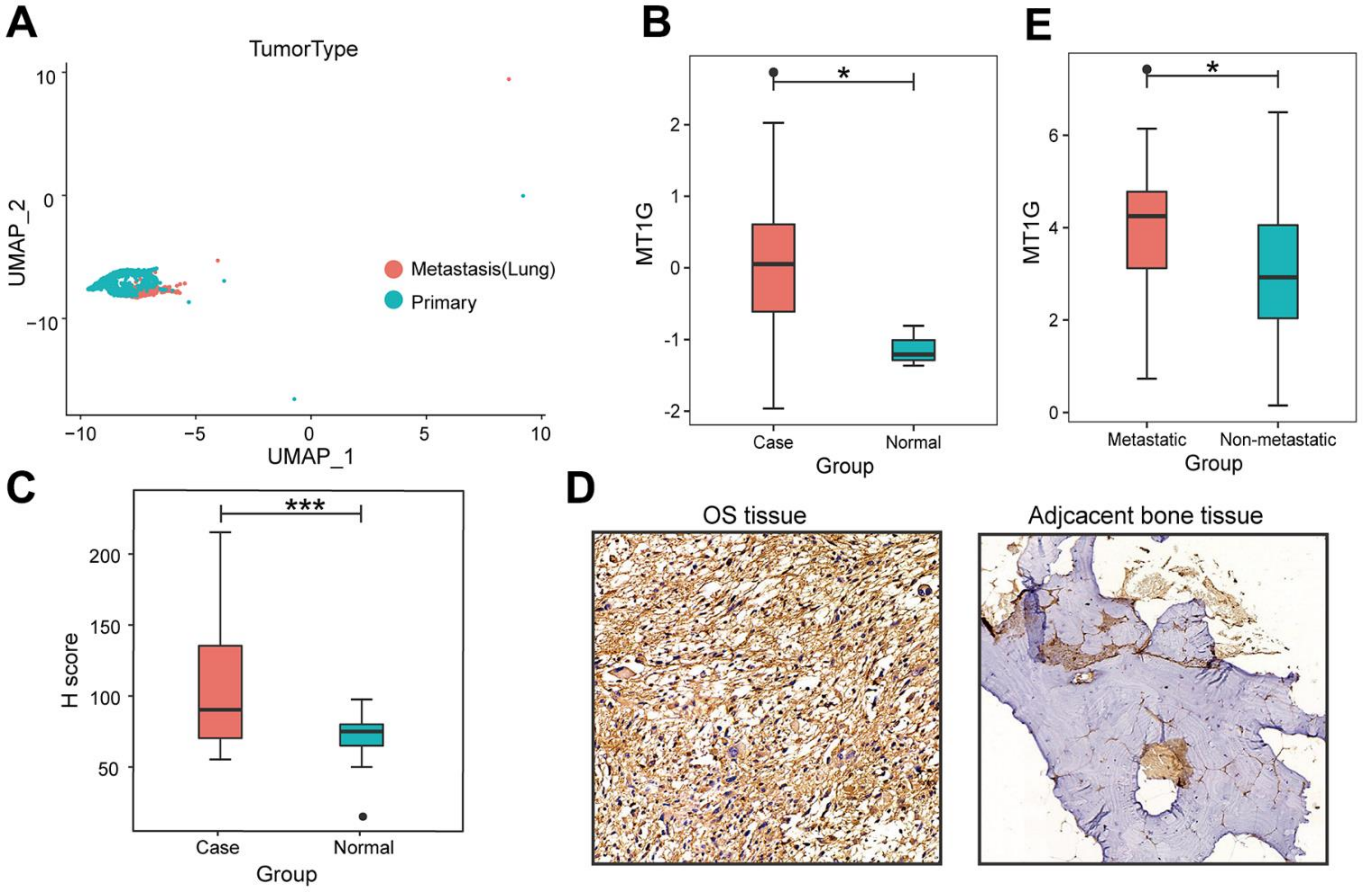
**Characterization of single-cell RNA-seq profiles.** (**A**) The clustering result of 2011 MSCs using the UMAP nonlinear dimensional reduction method colored by tissue origin. (**B**) MT1G was obviously more highly expressed in tumor tissues than in normal tissues. (**C**) In contrast to the primary tumor tissues, MT1G was obviously more highly expressed in metastatic tumor tissues. (**D**) Quantification of MT1G IHC staining in OS tissues (n=13) and adjacent cancerous tissues (n=13). (**E**) High/low H score of MT1G ICH images. (ns, p >0.05; *p <0.05; **p <0.01; ***p <0.001).

The flowchart demonstrates the overall design and process about this study (Figure 1). The variance analysis revealed the top 10 significantly DEGs across the cell samples (Supplementary Figure 1A). The principal component analysis (PCA) method screened the significantly correlated genes in each component. The top 30 significantly correlated genes were shown via heatmap and dot plot in Supplementary Figure 1A, 1B. The first 35 PCs represented the main differences between the cells (Supplementary Figure 1B). According to the UMAP algorithm and marker gene annotation, 2011 MSCs were identified in metastatic tissue and primary tissue (Figure 2A). A total of 7012 DEGs between metastatic tissue and primary tissue of MSCs were identified. We downloaded the OS bulk data of GSE33382 and conducted differential expression analysis. The results showed that MT1G was highly expressed in tumor tissues compared with normal tissues (Figure 2B). Furthermore, MT1G was more highly expressed in metastatic tumor tissues than in primary tumor tissues (Figure 2C). We validated MT1G expression using 13 OS tissues and 13 adjacent cancerous tissues as the validation dataset. MT1G expression levels were confirmed using IHC (Figure 1D, 1E).

### Identification of DEGs

**Figure 3:**
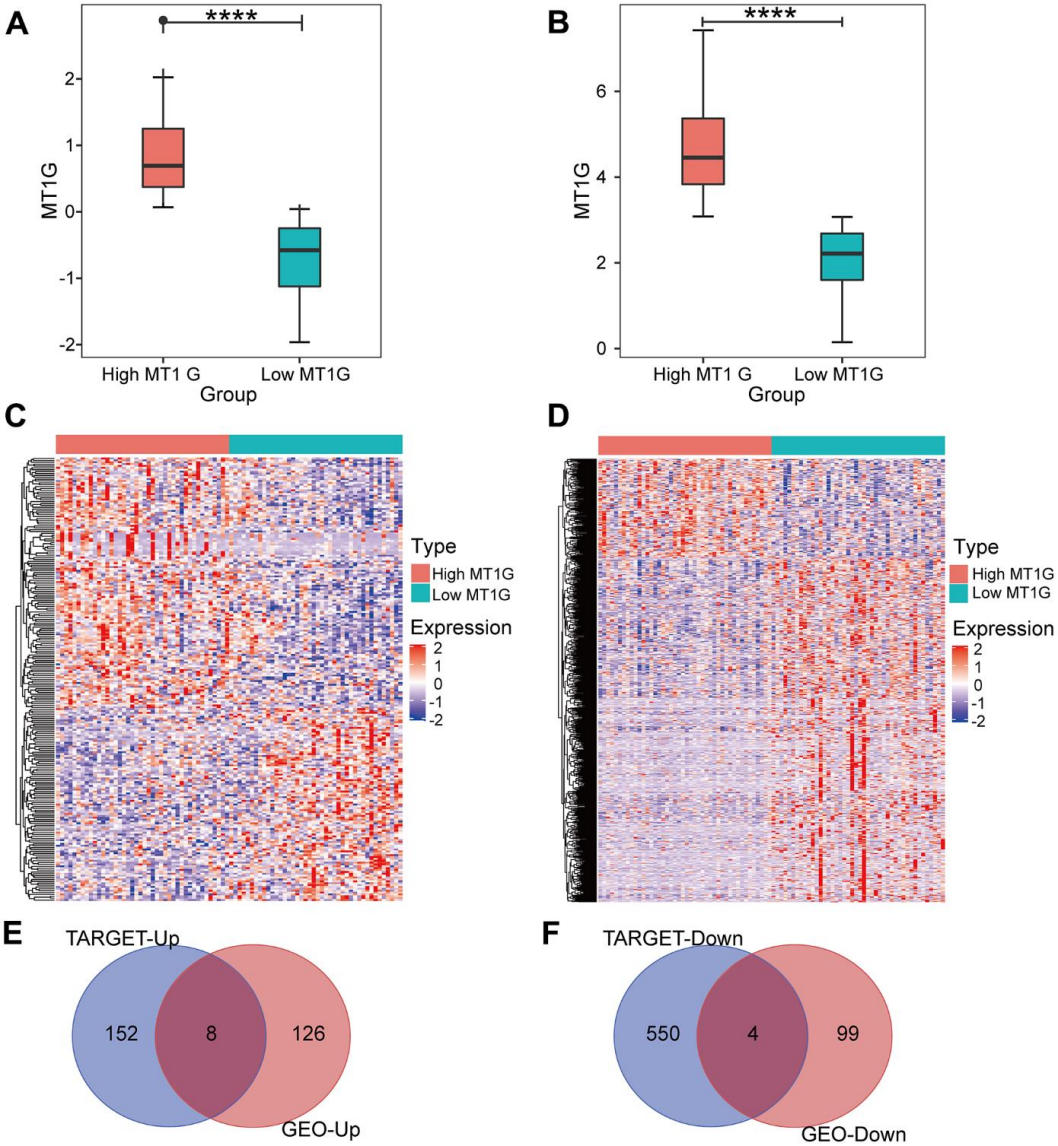
**Expression analysis for MT1G and coexpressed genes.** (**A**) MT1G expression in the GSE33382 cohort. (**B**) MT1G expression in the TARGET cohort. (**C**) The median expression level of MT1G in OS samples in the GSE33382 data set was used to divide patients into high/low expression groups, and the significant DEGs between the two groups were displayed in the form of a heatmap. (**D**) The median MT1G expression value of OS samples in the TARGET data set was used to sort the patients into high/low expression groups, and the significant DEGs between the two groups were displayed in the form of a heatmap. (**E**) Venn diagram of the intersection of upregulated DEGs in the GEO and TARGET datasets. (**F**) Venn diagram of the intersection of downregulated DEGs in the GEO and TARGET datasets. (ns, p >0.05; *p <0.05; **p <0.01; ***p <0.001).

After screening for DEGs between high and low MT1G-expression groups (Figure 3A), we found 134 upregulated genes and 103 downregulated genes from the high and low MT1G expression groups (Supplementary Table 1), for a total of 237 coexpressed genes (Figure 3C). The TARGET cohort was subsequently divided into high/low MT1G expression groups using the same strategy (Figure 3B), and 160 upregulated genes and 557 downregulated genes were screened (Supplementary Table 2 and Figure 3D). Finally, we screened 12 common DEGs (8 upregulated and 4 downregulated) from the two DEG groups (Figure 3E, 3F).

### Functional enrichment analysis and GSEA

**Figure 4:**
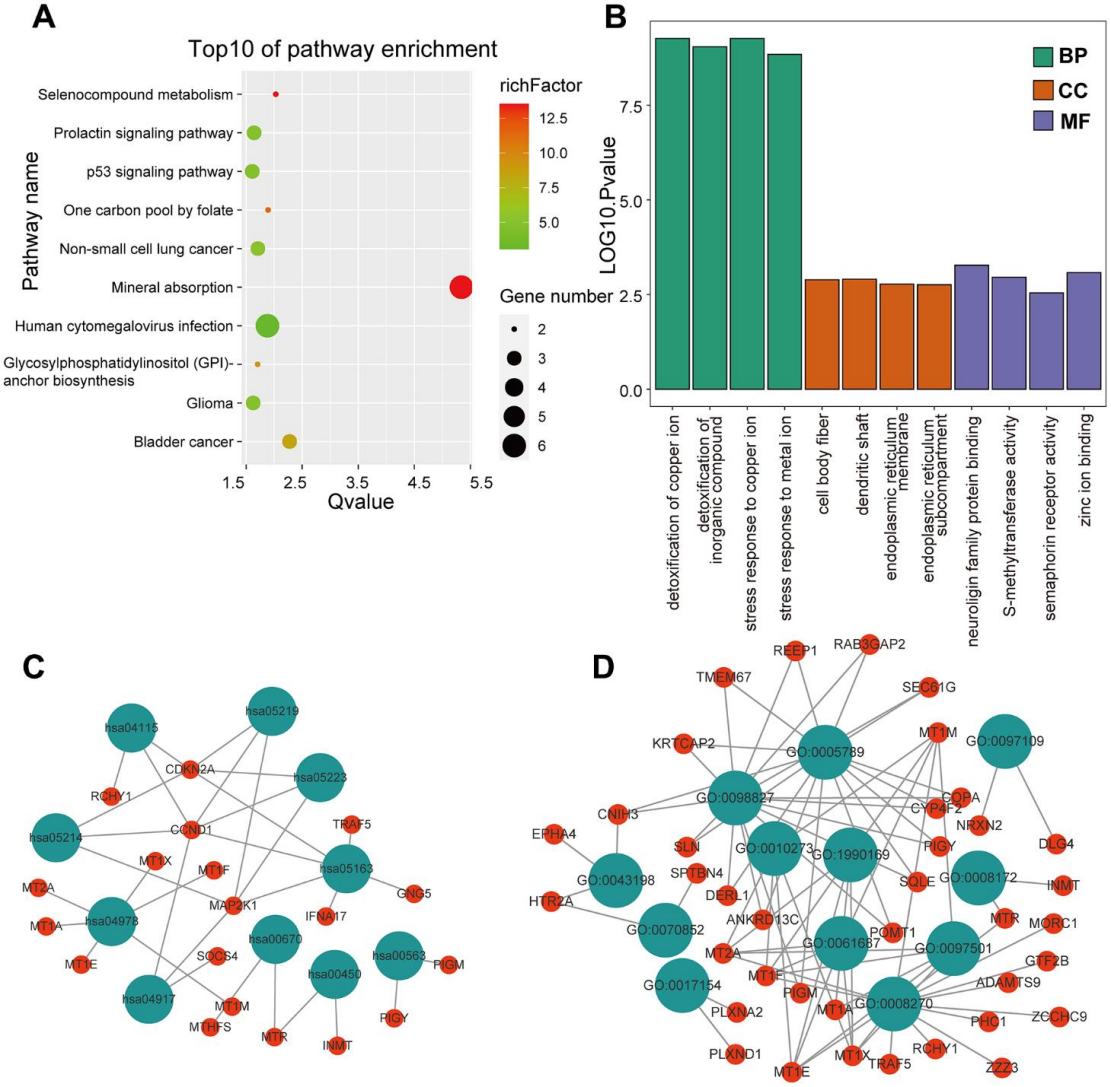
**GO and KEGG enrichment analysis in the GEO cohort.** (**A**) The bubble graph package was applied to visualize the results of KEGG enrichment analysis; the bubble size represented the number of enriched genes, and the color represented the enrichment ratio in the GSE33382 data set. (**B**) The bar graph for GO enrichment analysis; length represented significance in the GSE33382 data set. (**C**) A functional enrichment network based on KEGG analysis of the GSE33382 data set. (**D**) A functional enrichment network based on GO analysis of the GSE33382 data set.

**Figure 5:**
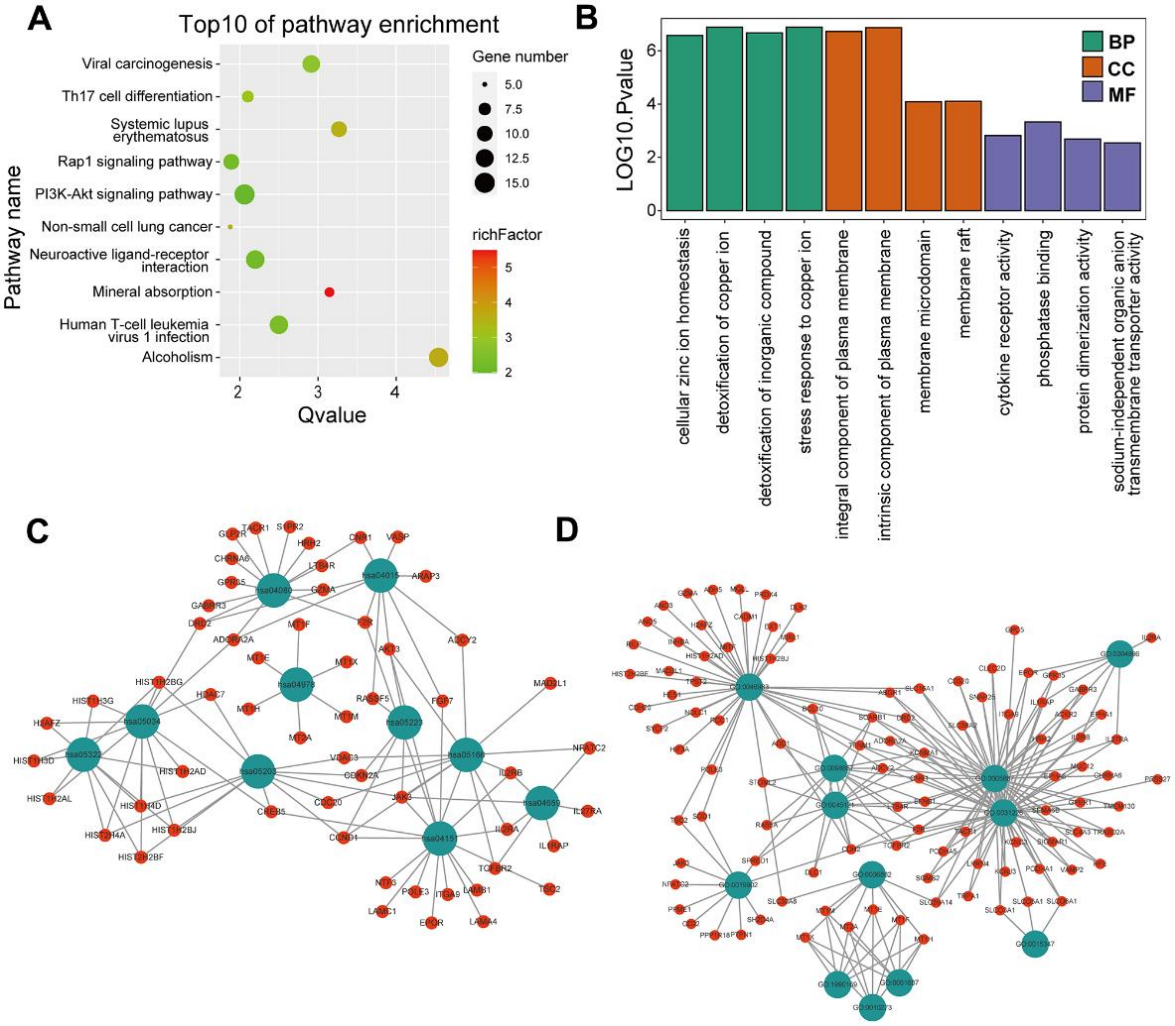
**GO and KEGG enrichment analyses in the TARGET cohort.** (**A**) The bubble graph package was applied to visualize the results of KEGG enrichment analysis; the bubble size represented the number of enriched genes, and the color represented the enrichment ratio in the TARGET data set. (**B**) The bar graph for GO enrichment analysis; length represented significance in the TARGET data set. (**C**) A functional enrichment network based on KEGG analysis of the TARGET data set. (**D**) A functional enrichment network based on GO analysis of the TARGET data set.

**Figure 6:**
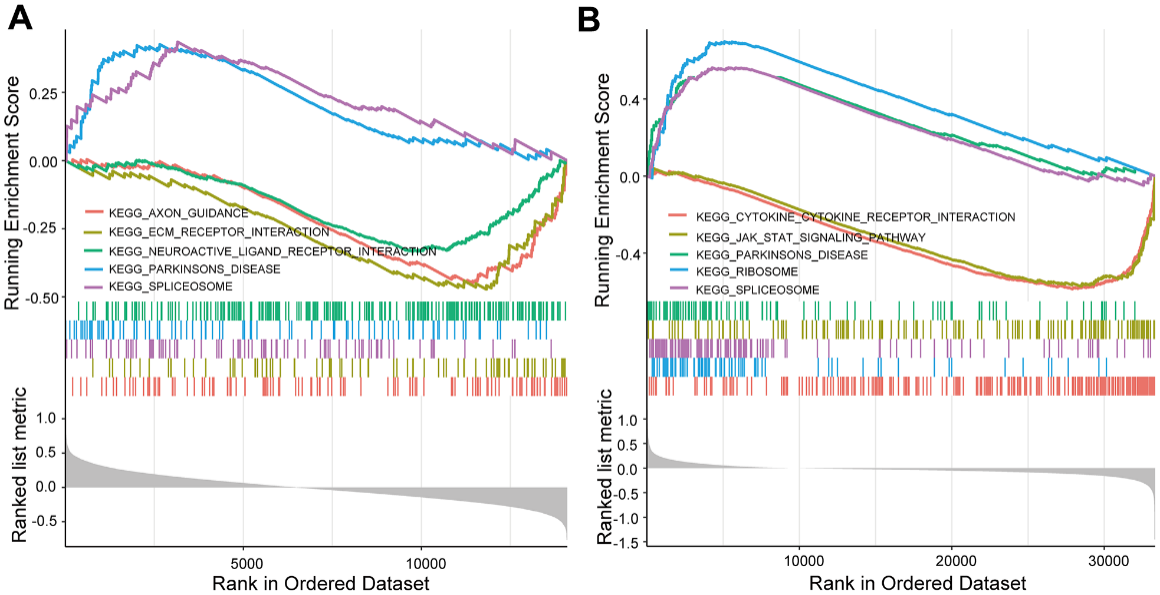
**GSEA enrichment analysis.** (**A**) GSEA enrichment analysis in the GEO cohort. (**B**) GSEA enrichment analysis in the TARGET cohort.

KEGG and GO enrichment results showed that the core genes were mainly enriched in the following pathways: the p53 signaling pathway, human cytomegalovirus infection, tumor-associated pathways, detoxification of copper ions, and dendritic shaft and neuroligin family protein binding (Figure 4 and Supplementary Tables 3–6). The enriched terms for the TARGET cohort were the PI3K-Akt signaling pathway, Rap1 signaling pathway, copper ion detoxification, intrinsic components of the plasma membrane and phosphatase binding. (Figure 5 and Supplementary Tables 7–10). We selected c2.cp.kegg.v7.4.entrez as the reference gene set for our GSEA. In the GEO cohort, DEGs mainly involved spliceosome and ECM receptor interactions (Figure 6A). In the TARGET cohort, DEGs were involved in spliceosome, ribosome and cytokine-cytokine receptor interactions (Figure 6B).

### Construction of PPI networks and hub-gene screening

**Figure 7:**
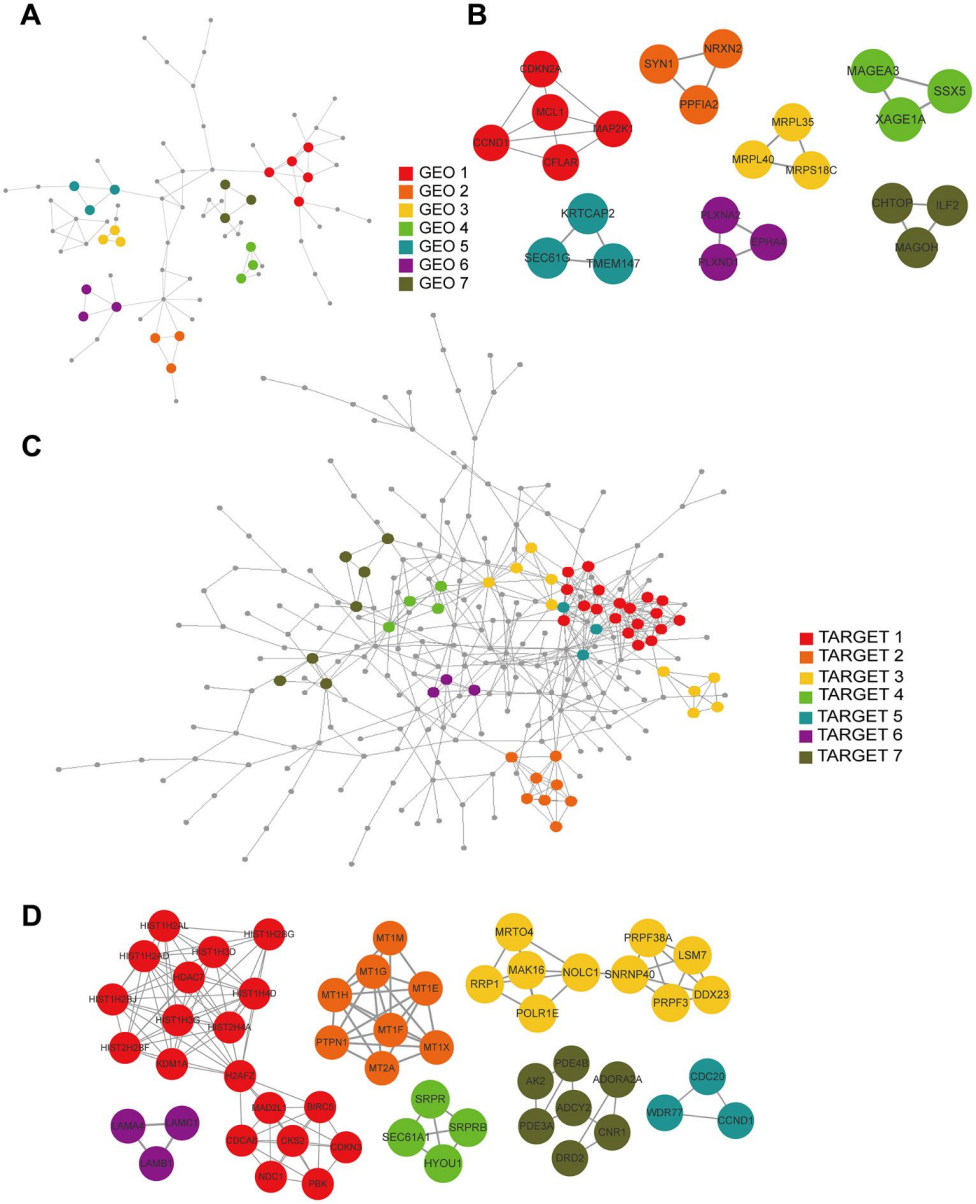
**PPI and hub gene cluster network construction.** (**A**) A PPI network from the GSE33382 data set. (**B**) Seven hub gene clusters obtained by the MCODE clustering algorithm from the GSE33382 data set. (**C**) A PPI network from the TARGET data set. (**D**) Seven hub gene clusters obtained by the MCODE clustering algorithm from the TARGET data set.

We constructed PPI networks from the GEO and TARGET cohort DEGs separately using the STRING tool (Figure 7A, 7C) and used the MCODE clustering algorithm to filter hub gene clusters. For the GEO cohort, screened hub genes were linked in the following networks: MCODE_1 (CCND1, MAP2K1, CDKN2A, MCL1, CFLAR), MCODE_2 (SYN1, PPFIA2, NRXN2), MCODE_3 (MRPL35, MRPS18C, MRPL40), MCODE_4 (MAGEA3, XAGE1A, SSX5), MCODE_5 (TMEM147, KRTCAP2, SEC61G), MCODE_6 (EPHA4, PLXND1, PLXNA2) and MCODE_7 (ILF2, CHTOP, MAGOH; Figure 7B). Hub genes in the TARGET cohort were involved in the following networks: MCODE_1 (BIRC5, CDCA8, CDKN3, CKS2, H2AFZ, HDAC7, HIST1H2AD, HIST1H2AL, HIST1H2BG, HIST1H2BJ, HIST1H3D, HIST1H3G, HIST1H4D, HIST2H2BF, HIST2H4A, KDM1A, MAD2L1, NDC1, PBK), MCODE_2 (MT1E, MT1F, MT1G, MT1H, MT1M, MT1X, MT2A, PTPN1), MCODE_3 (DDX23, LSM7, MAK16, MRTO4, NOLC1, POLR1E, PRPF3, PRPF38A, RRP1, SNRNP40), MCODE_4 (HYOU1, SEC61A1, SRPR, SRPRB), MCODE_5 (CCND1, CDC20, WDR77), MCODE_6 (LAMA4, LAMB1, LAMC1), and MCODE_7 (ADCY2, ADORA2A, AK2, CNR1, DRD2, PDE3A, PDE4B; Figure 7D).

### Expression analysis of MT1G-related genes

**Figure 8:**
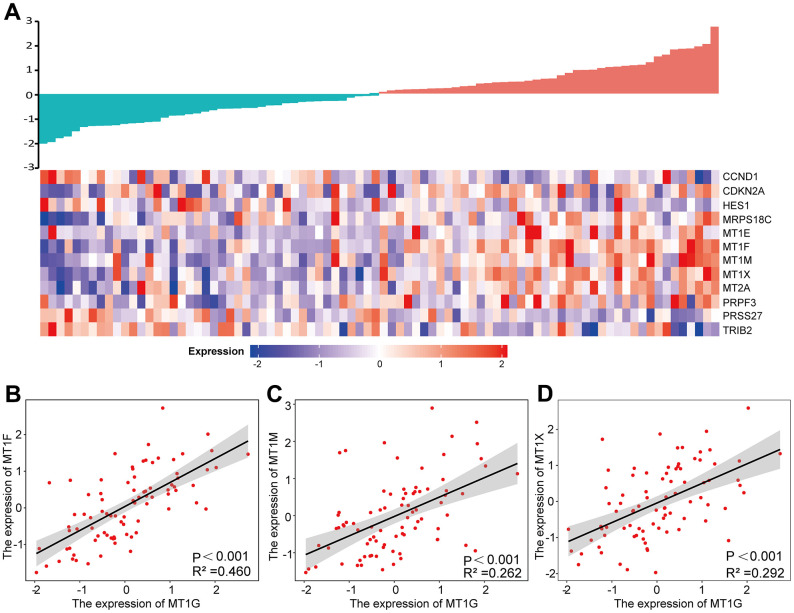
**Expression analysis of MT1G-related genes.** (**A**) A gene expression-related heatmap of the 12 common DEGs between GEO and TARGET datasets. (**B**–**D**) Expression correlations between MT1F, MT1M and MT1X and MT1G were shown as scatter plots. p <0.05 was set for significance.

By comparing the 12 common DEGs in the GEO and TARGET cohorts, we found that the expression of the MT1F, MT1M and MT1X genes was correlated with the expression of the MT1G gene (Pearson coefficient >0.5) (Figure 8A). The associations between the above genes and MT1G expression were shown in Figure 8B–8D.

### ceRNA network

**Figure 9:**
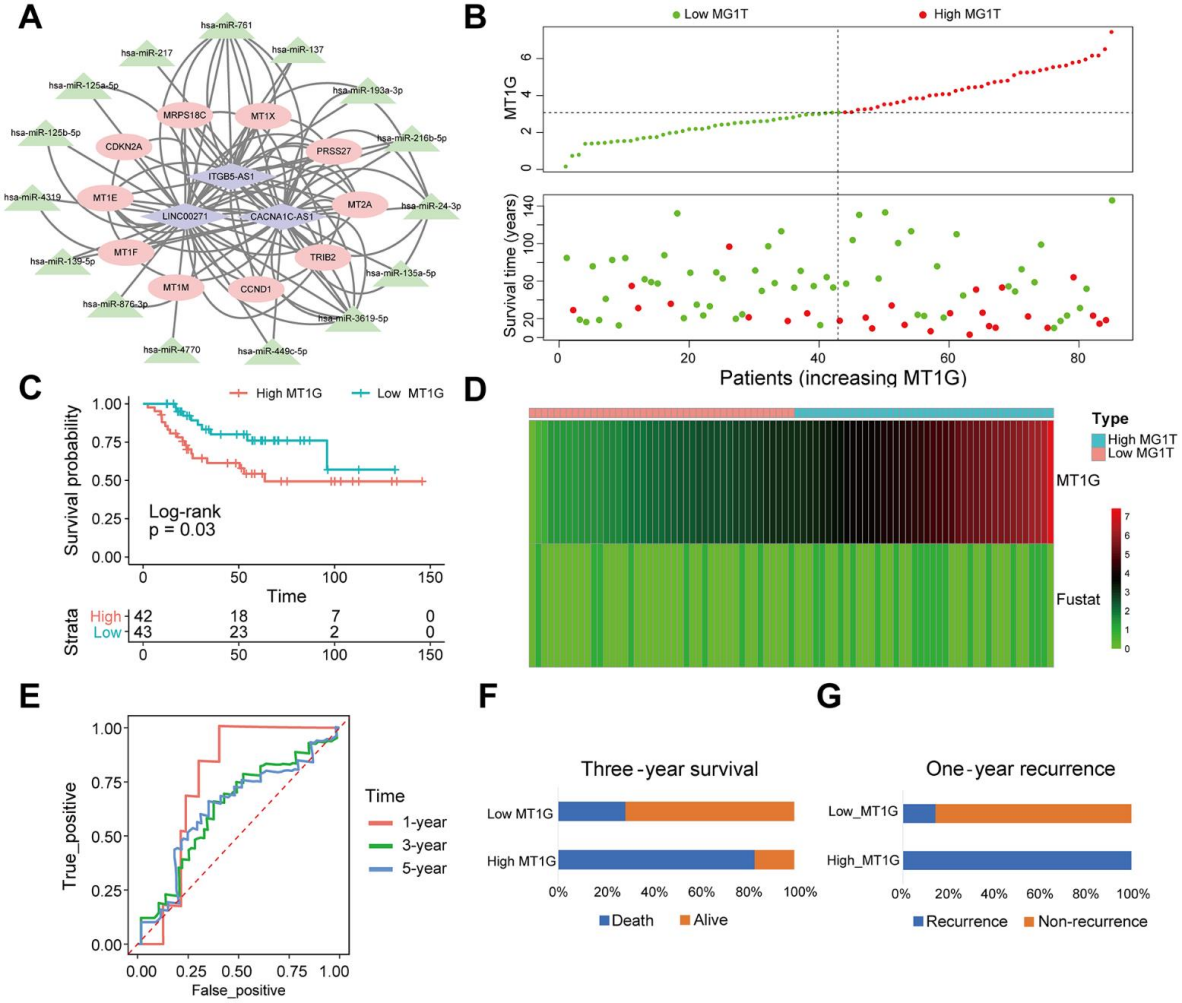
**MT1G-related ceRNA network construction and survival analysis.** (**A**) A ceRNA network: blue diamond, lncRNAs; green triangle, miRNAs; red round, mRNAs. (**B**) Scatter plot of the expression of MT1G and OS patients’ survival time. (**C**) The KM curves of the high- and low-MT1G expression groups (**D**) Heatmap of the expression of MT1G and OS patients’ survival time. (**E**) Time-dependent ROC curve of MT1G expression in predicting OS overall survival. (**F**) Three-year survival of 13 OS patients with high or low MT1G expression. (**G**) One-year recurrence of 13 OS patients with high or low MT1G expression. (ns, p >0.05; *p <0.05; **p <0.01; ***p <0.001).

We obtained 3 DELs from the TARGET database using limma, including LINC00271, CACNA1C-AS1 and ITGB5-AS1. MiRcode websites were subsequently used to predict miRNAs that were highly consistent with the DELs. Furthermore, we identified mRNAs targeted by the miRNAs through a series of databases and then constructed a ceRNA network with DEGs (Figure 9A).

### Validating the prognostic value of MT1G expression in OS

The results of survival analysis in different MT1G expression groups showed that high expression of MT1G was associated with poor prognosis and increased risk of death in OS patients (Figure 9B, 9C). The MT1G high expression group had a shorter survival time than the MT1G low expression group (Figure 9D). Time-related ROC curves showed that the expression level of MT1G could predict the survival times of OS patients. The area under the curve values were 0.756, 0.627, and 0.627 for 1-year, 3-year, and 5-year survival, respectively (Figure 9E). In the validation cohort, OS patients with high MT1G expression had significantly lower 3-year survival and 1-year recurrence rates than those who had low MT1G expression (Figure 9F, 9G).

## DISCUSSION

OS is a primary bone cancer commonly found in long bones of children and adolescents [23]. OS is characterized by its high malignancy, leading to irregular bone growth and an impaired immune response in patients. OS cells mainly metastasize to the lungs and liver. Currently, the main treatment modalities for OS comprise preoperative chemotherapy, surgical resection, and postoperative chemotherapy. Regrettably, the above treatment strategies demonstrate effectiveness only in patients with localized OS, whereas those with advanced or metastatic OS often encounter chemotherapy resistance, leading to unfavorable treatment outcomes and a grim prognosis [24]. The overall five-year survival rate is approximately 65% for patients with local OS and only 20% for patients with metastatic OS [25]. The challenging nature of OS treatment arises from its impact on diverse patient populations and the complex genetic alterations involved [26].

Metallothioneins (MTs) are intracellular cysteine-rich proteins characterized by low molecular weight. Previous studies have demonstrated their association with aggressive phenotypes and treatment resistance in various cancers. Among MTs, MT1 has been shown to affect tumor growth. For example, Wang Y et al. showed that MT1G can inhibit proliferation and invasion or induce apoptosis [27]. The effects of MT1G differ among different cancers. Therefore, we focused on analyzing its biological function in OS in this study.

MT1G exhibited high expression in both situ BMSCs and metastatic bone metastases, and high expression was associated with a poor prognosis. Previous studies have indicated that MT1G is involved in sorafenib resistance through the inhibition of a novel form of regulated cell death known as ferroptosis [20]. MT1G-overexpressing cells showed a significant reduction in cell death when exposed to cytotoxins such as endostatin [28]. Therefore, we speculate that MT1G may be a potential therapeutic target based on its role in MSCs. In our study, we found that MT1G is enriched in certain cancer pathways as well as cytokine signaling pathways, with a typical representative being the PI3K-Akt signaling pathway, which is a pro-mitotic signaling pathway [29], and it is also associated with angiogenesis [30].

We searched for genes co-expressed with MT1G to determine the expression pattern of MT1G in OS. We found that the expression of MT1F and MT1X genes is positively correlated with the expression of the MT1G gene. Studies have shown that MT1F is associated with poor clinical outcomes in non-small cell lung cancer [31]. In thyroid cancer, compared to malignant cells of the primary tumor, the characteristic of lymph node metastatic cells is the upregulation of MT1X and MT1G [32]. These findings also suggest the potential value of genes co-expressed with MT1G, which could be used for clinical prognosis.

Effective novel biomarkers are of great help in selecting treatment strategies for OS [33]. We found that high expression of MT1G is associated with poor prognosis and increased risk of death in OS patients, and ROC curve analysis showed that the expression level of MT1G can predict the survival time of OS patients. This was also validated in the validation cohort. In conclusion, this study confirms the prognostic value of MT1G in OS.

## Supplementary Material

Supplementary Figure 1

Supplementary Table 1

Supplementary Table 2

Supplementary Table 3

Supplementary Table 4

Supplementary Table 5

Supplementary Table 6

Supplementary Table 7

Supplementary Table 8

Supplementary Table 9

Supplementary Table 10
