# Supplementary material for: Bioinformatics analysis and validation of mesenchymal stem cells related gene MT1G in osteosarcoma
Source: Aging (Albany NY). 2024 May 13;16(9):8155–70. doi: 10.18632/aging.205809 (PMC11131992; doi:10.18632/aging.205809)
Supplement: Supplementary Figure 1 [file aging-16-205809-s001.pdf]

## SUPPLEMENTARY FIGURE

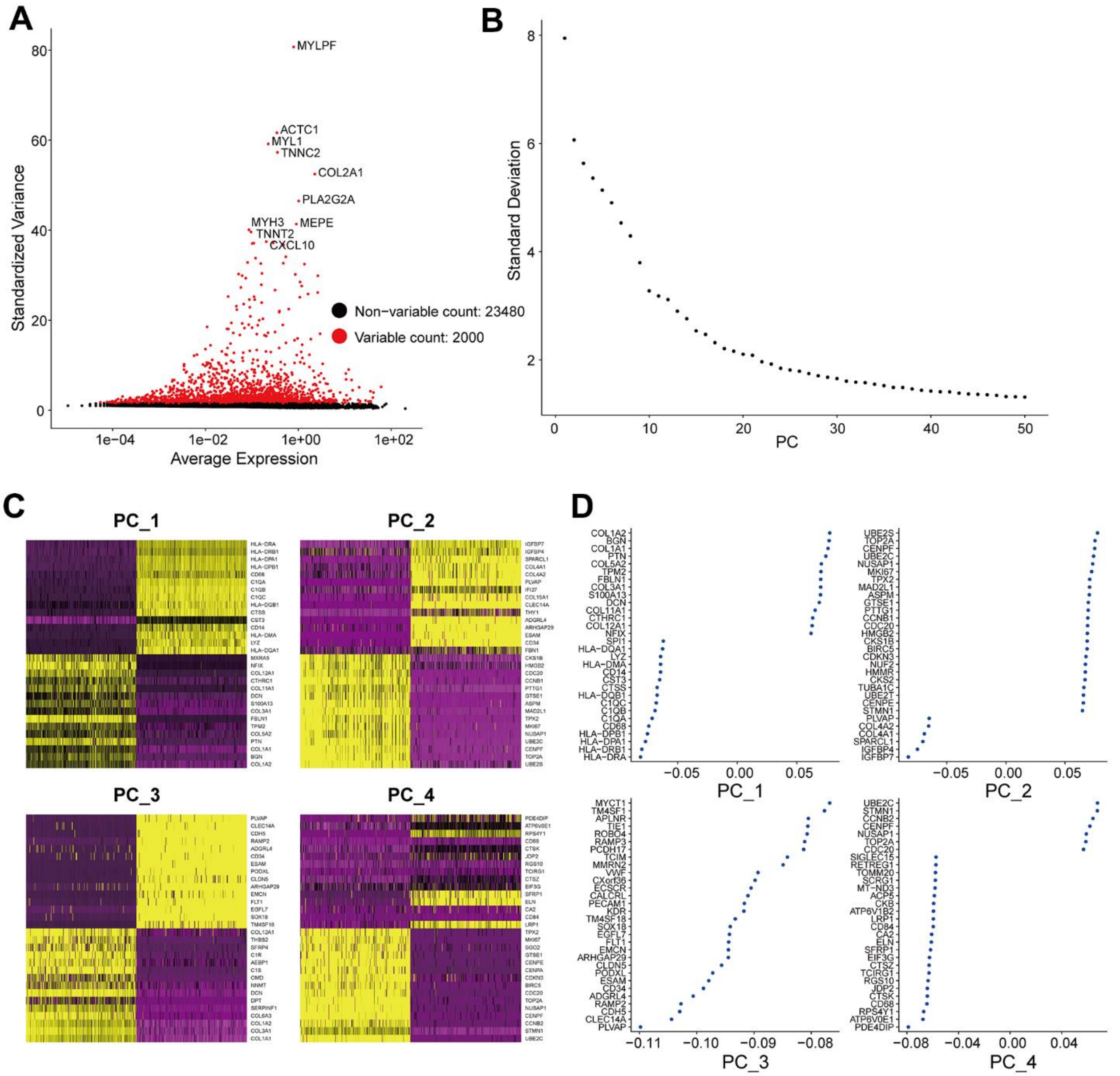


**Supplementary Figure 1. Processing of single-cell RNA-seq data.** (**A**) The characteristic variance diagram of the gene symbols. Each spot represented a gene. Red spots represented highly variable genes with significant differences across cells. (**B**) Principal component sorting elbow graph based on the percentage of variance explained by each one. (**C**) Cluster analysis across each component. The colors ranging from purple to golden yellow represented the expression levels of correlated genes from low to high. (**D**) Correlation analysis of the most altered genes in each component.
